# Thrombospondin-1 in Chronic Kidney Disease Driven Cardiac Dysfunction: More Than Just a Biomarker?∗

**DOI:** 10.1016/j.jacbts.2024.03.008

**Published:** 2024-05-27

**Authors:** Attila Kiss

**Affiliations:** Ludwig Boltzmann Institute for Cardiovascular Research at the Center for Biomedical Research and Translational Surgery, Medical University of Vienna, Vienna, Austria

**Keywords:** cardiorenal syndrome, chronic kidney disease, HFpEF, left ventricular hypertrophy, thrombospondin 1

**Figure:**
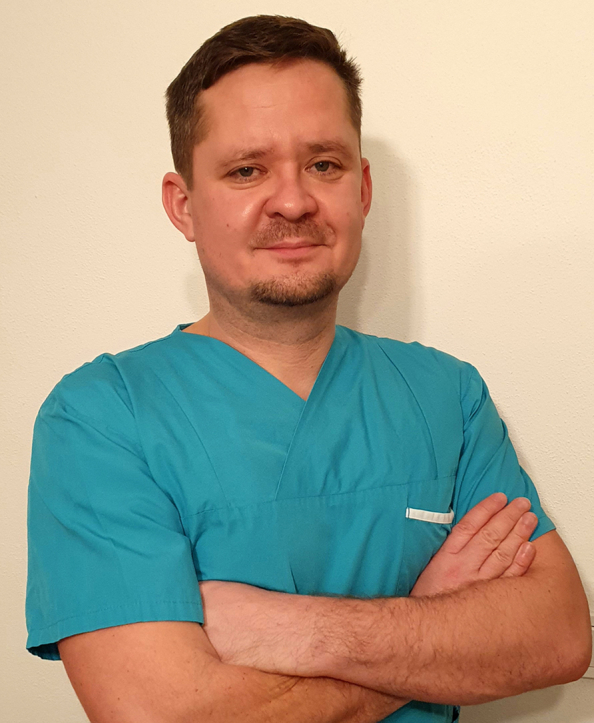

Chronic kidney disease (CKD) affects 15% to 20% of adults globally and increases the risk of various cardiovascular diseases (CVDs); consequently, cardiorenal syndromes encompasses conditions in which failure of either the kidneys or the heart leads to failure of the other organ.^1^ In general, progressive deterioration of the left ventricular (LV) pump function leads to insufficient perfusion of the kidney and renal dysfunction entails increased blood pressure, fluid retention, and elevation of uremic toxins levels in the circulation, which eventually foists increased workload on the heart and results in cardiomyocyte hypertrophy, stiffness, metabolic alterations, and extracellular matrix (ECM) remodeling with concomitant cardiac fibrosis.^1^ Current treatments for chronic CKD involve lifestyle changes, medication, and dialysis, which can delay disease progression but they do not usually rescue the adverse cardiac remodeling. Consequently, CKD-driven cardiac dysfunction is still largely incurable; therefore, there is an urgent need for evidence-based therapies to identify, treat, and significantly improve both the cardiovascular and kidney dysfunction among patients with CKD.

To better understand the progression of cardiovascular dysfunction in CKD, and eventually reduce the premature mortality in patients with CKD, it is also important to identify one or likely more highly sufficient/selective circulating biomarkers. Accordingly, previous studies summarized the use of serum biomarkers (eg, atrial and B-type natriuretic peptide, isoforms of troponins, adiponectin, plasma growth differentiation factor-15, ECM proteins) for cardiovascular disease risk prediction in CKD.^2^^,^^3^ Most cardiovascular diseases (eg, CKD) involve severe remodeling of the ECM, culminating in the formation of fibrotic tissue that is deleterious to organ function and ECM protein may serve as a biomarker for disease progression^4^ and targeted therapy for heart failure in CKD. In addition, the accumulation of non-hemodialyzable uremic toxins, such as indoxyl sulfate and p-cresyl sulfate, in the circulation and in tissues serves as a biomarker and is associated with the progression of CKD, and CVD in patients with CKD.^5^

The ECM network is composed mainly of collagen, which provides a scaffold for the cellular constituents of the heart and, furthermore, contributes to the effective transmission of the contractile force. Collagen type I and type III are highly abundant in the heart, and responsible for the elasticity of the matrix network as well as cardiomyocyte stiffness and mechanical compliance. Besides collagens, the ECM also includes nonstructural proteins such as glycosaminoglycans, proteoglycans, and glycoproteins. However, despite its high clinical significance, the exact underlying signaling mechanisms of cardiac ECM remodeling, fibrosis still remains elusive in CKD.

A recent study in this issue of *JACC: Basic to Translational Science* by Julovi et al^6^ demonstrated for the first time that the disruption of ECM glycoprotein thrombospondin (TSP)-1, which in general may be secreted by various cells, including cardiomyocytes, fibroblasts, and smooth muscle cells in the myocardium, provides cardioprotection in a mouse model of CKD.

When extrapolating from preclinical (animal study and using human cardiomyocytes) findings to patients with CKD, the authors also issue the differential protein levels of circulating TSP-1 and myocardial gene expression of TSP-1 among patients with CKD in comparison with healthy patients, consequently higher levels of TSP-1 in plasma associated with a lower LV ejection fraction and an increase in LV mass index, respectively. Surprisingly, TSP-1 knockout mice do not show renal protection, despite its detrimental role in renal fibrosis^7^ and ischemic acute kidney injury. Furthermore, TSP-1 upregulation in the kidneys is associated with aging, and subsequently renal dysfunction.^7^ Accordingly, CKD and CVD are common in older people, and their prevalence increases in parallel with age. This is a very important and thought-provoking issue with regard to the findings of Julovi et al,^6^ and one can speculate whether TSP-1 upregulation in plasma may reflect renal failure rather than cardiomyocyte dysfunction/cardiac ECM remodeling in patients with CKD. Consequently, using renal tissue–specific TSP-1 knockout mice with advanced age theoretically poses an attractive alternative to clarifying the unrealized renal benefits of TSP-1 downregulation and simultaneously raise awareness of the progression of CKD-driven LV hypertrophy and dysfunction.

One of the greatest fundamental findings by Julovi et al^6^ was to recognize the molecular signaling mechanism that mitigates TSP-1 upregulation in cardiomyocytes. Accordingly, nonhemodialyzable uremic toxins, for example, indoxyl sulfate binds to aryl hydrocarbon receptor in cardiomyocytes, a ligand activated transcription factor that may be involved in the regulation of TSP-1. Consequently, its overexpression resulted in cardiomyocyte hypertrophy and diastolic dysfunction in association with cardiac fibrosis in a mouse model of CKD and isolated human cardiomyocytes, respectively. Consistent with that finding, the observed impaired LV function and hypertrophy are associated with plasma indoxyl sulfate upregulation in a rat model of CKD.^8^ Although not explicitly stated in the report, we raise the question of whether indoxyl sulfate solely regulates the expression of TSP-1 in cardiomyocytes or whether its overexpression also contributes to the enhancement of pro-inflammatory and pro-fibrotic mechanisms in different cells types in the heart tissue? The authors did not provide further evidence or investigate why the high levels of indoxyl sulfate even in TSP-1 knockout mice still resulted in a strong cardioprotection. A question that emerges from these findings is whether and to what extent the TSP-1 plays a role in cardiac dysfunction in CKD, whether TSP-1 in cardiomyocytes affects the phenotypes of neighboring cells and microenvironment (eg, in cardiac fibroblasts). Indeed, cardiomyocytes are critical contributors to the myocardial fibrotic programs,^9^ in response to injurious stimuli (mechanical stress, metabolic dysfunction, and inflammatory cytokines) that might induce the cardiac fibroblast activation (fibroblasts to myofibroblast transition) and eventually cardiac fibrosis as well as cardiomyocyte stiffness.

A final question addressed by the present study^6^ is whether TSP-1 might be a trigger for senescence in the heart and demonstrated that senescence and pro-inflammatory markers in the heart tissue were mitigated in TSP-1 knockout mice with CKD. In line with that, differentially expressed gene expression analysis in the human dataset also confirmed the potential role of senescence in the progression of CKD and the risk for CVD in CKD. These findings raise the question of the probability of senolytic treatment^10^ to alleviate the effects of TSP-1 in adverse cardiac remodeling in CKD.

In summary, the findings of Julovi et al^6^ provide a first piece of evidence that a novel signaling pathway drives uremic toxin; for example, indoxyl sulfate-induced cardiomyocyte dysfunction through the upregulation of the ECM protein TSP-1. These findings were also confirmed in patients with CKD, lending support to the premise that further stimulation of testing monoclonal antibodies or inhibitors that block the actions of TSP-1. However, further multicenter, randomized clinical trials are warranted to confirm the interaction between indoxyl sulfate and TSP-1 and to prove evidence that TSP-1 levels are gradually increasing in CKD and CKD-driven cardiac hypertrophy and diastolic dysfunction.

## Funding Support and Author Disclosures

The author has reported that he has no relationships relevant to the contents of this paper to disclose.
